# Expression of an Efficient Selection Marker Out of a Duplicated Site in the ITRs of a Modified Vaccinia Virus Ankara (MVA)

**DOI:** 10.3390/vaccines12121377

**Published:** 2024-12-06

**Authors:** Sirine Abidi, Aurora Elhazaz Fernandez, Nicole Seehase, Lina Hanisch, Alexander Karlas, Volker Sandig, Ingo Jordan

**Affiliations:** 1 ProBioGen AG, 13086 Berlin, Germany; sirine.abidi@probiogen.de (S.A.); aurora.elhazazfernandez@mdc-berlin.de (A.E.F.); nicole.seehase@tentamus.com (N.S.); alexander.karlas@probiogen.de (A.K.); volker.sandig@probiogen.de (V.S.); 2 Berlin Institute for Medical Systems Biology (BIMSB), 10115 Berlin, Germany; 3 Tentamus Pharma & Med Deutschland GmbH, 76149 Karlsruhe, Germany

**Keywords:** inverted terminal repeat, vaccinia, MVA, innate immune system, tetherin

## Abstract

**Background/Objectives**: Poxviruses are large DNA viruses that replicate in the host cytoplasm without a nuclear phase. As vaccine vectors, they can package and express large recombinant cassettes from different positions of their genomic core region. We present a comparison between wildtype modified vaccinia Ankara (MVA) and isolate CR19, which has significantly expanded inverted terminal repeats (ITRs). With this expansion, a site in wildtype MVA, called deletion site (DS) IV, has been duplicated at both ends of the genome and now occupies an almost central position in the newly formed ITRs. **Methods**: We inserted various reporter genes into this site and found that the ITRs can be used for transgene expression. However, ITRs are genomic structures that can rapidly adapt to selective pressure through transient duplication and contraction. To test the potential utility of insertions into viral telomers, we inserted a factor from the cellular innate immune system that interferes with viral replication as an example of a difficult transgene. **Results**: A site almost in the centre of the ITRs can be used for transgene expression, and both sides are mirrored into identical copies. The example of a challenging transgene, tetherin, proved to be surprisingly efficient in selecting candidate vectors against the large background of parental viruses. **Conclusions**: Insertion of transgenes into ITRs automatically doubles the gene doses. The functionalisation of viruses with tetherin may accelerate the identification and generation of recombinant vectors for personalised medicine and pandemic preparedness.

## 1. Introduction

The *Poxviridae* are a diverse family with insects and vertebrates as hosts. The fully matured enveloped virions are ovoid in shape with up to 300 nm in length and contain a linear DNA genome of 128 to 365 kb that codes for more than 200 genes [1]. Recombinant poxviruses are genetically and physicochemically stable vectors that can be designed to express multiple large transgenes for immunotherapy and heterologous vaccination strategies [2]. Modified vaccinia Ankara (MVA) is an attenuated poxvirus that has been obtained by serial adaptation of a replication-competent smallpox vaccine strain to chicken embryo fibroblasts [3]. MVA has lost approximately 15% of its genome at six major deletion sites (DS I through VI) that together with accumulated additional minor disruptions and fragmentations in several genes contribute to the restricted host range [4,5,6]. In contrast to the parental vaccinia virus, MVA replication in mammalian cells is usually blocked or severely impaired [7,8,9].

**Figure 1:**
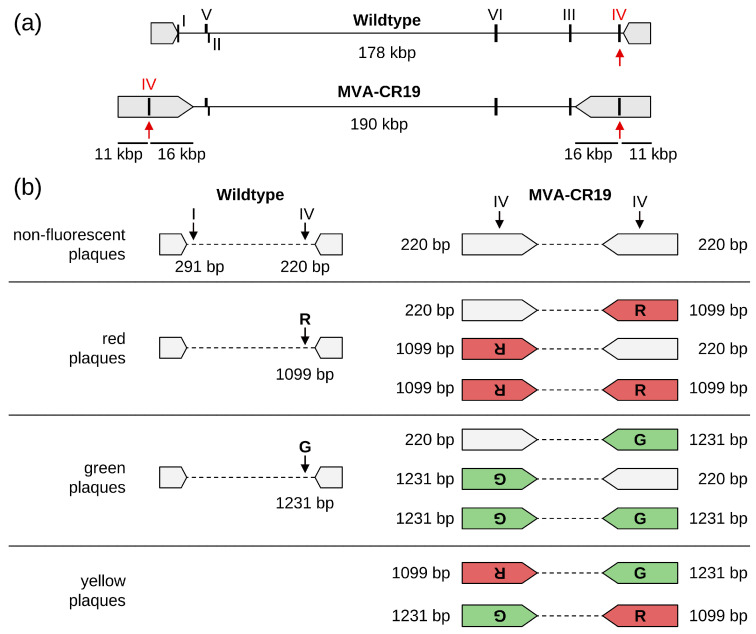
(**a**) Genomic organisation of WT MVA and strain CR19. Deletion sites I to VI are marked with Roman numerals and the inverted terminal repeats (ITRs) with grey arrow boxes. The ITRs have expanded in size in MVA-CR19 by a recombination event that has caused loss of DS I at the left side and duplication of DS IV (up-pointing red arrows) within the expanded ITRs at both sides of the genome. The distance from DS IV to the apex is 11 kb and to the core of the genome is 16 kb. (**b**) Possible genotypes and phenotypes for recombination of different markers into DS IV of WT MVA and MVA-CR19. Only the positions of DS I and DS IV are indicated with down-pointing arrows and roman numerals. The two ITRs are drawn as arrow boxes. DS IV is localised outside of the right ITR of WT and within both ITRs of MVA-CR19. DS I is localised outside of the left ITR of WT and is not present anymore in MVA-CR19. Grey arrow boxes are for non-modified ITRs. The letters in the filled arrow boxes of MVA-CR19 stand for the insertion of EGFP (G) or mCherry (R) into DS IV within the ITR. The bp numbers refer to the sizes of diagnostic PCR products obtained with the shown configuration using primers listed in Table S1.

We previously described a genetically stable variant of MVA (MVA-CR19) that is less dependent on cell-to-cell contacts for the spread of infectious units and replicates in single-cell suspension cultures [10,11]. At the genomic level, MVA-CR19 is characterised by three-point mutations in structural proteins and an expanded inverted terminal repeat (ITR) of 27 kb compared to the 10 kb of wildtype (WT) MVA [10]. The expansion was caused by a duplication of the right terminus in such a way that a significantly larger region is mirrored at both sides of the genomic DNA. Because of this rearrangement, DS I is lost in MVA-CR19 and DS IV is duplicated. The two DS IV loci are each positioned almost in the centre of the two newly formed ITRs: the distance of DS IV to the proximal terminus is 11 kb, and the distance to the end of the repeat and start of the genome core is 16 kb [10] (Figure 1a).

The deletion sites mark regions of the viral genome that are not essential for replication, and especially DS III is commonly used as a locus for insertion of transgene expression cassettes. Here, we were interested in testing the insertion of transgenes into a duplicated site, the DS IV of MVA-CR19, and also asked whether different transgenes can be inserted into the two sites that are separated by 169 kb. The answer to this question is not straightforward, as recombinant insertions into ITRs may disrupt signals that are important for genome replication and subsequent resolution of concatemers [12,13]. Poxviruses furthermore appear to adapt rapidly to selective pressures through the expansion and contraction of regions that encode accessory proteins such as host range factors, including those located in the ITRs [14,15,16,17,18]. The insertion of non-viral genes is expected to introduce transient regions of non-homology that may destabilise regions of the ITRs with unpredictable outcomes for the vaccine vector.

However, here, we present results that suggest that genes for fluorescent proteins can be stably inserted into the ITR and that equal copies are rapidly established on both sides of the genome. We have furthermore extended the study to assess effects beyond the reporter genes by inserting a challenging antigen—in this case, a novel selection marker, tetherin, a component of the innate immune system. This second objective of the study, the investigation of the expression of proteins that may burden viral replication, evolved into a surprisingly efficient protocol for the generation of recombinant MVA and may have applications in personalised medicine [19,20,21] and antigen screening for pandemic preparedness [22] based on poxviral vectors.

## 2. Materials and Methods

### 2.1. Design of the Shuttle Plasmids for DS IV

The shuttle vector pSh III ELP11 Dual for insertion of EGFP and mCherry into deletion site (DS) III has been described previously [10]. The shuttle vectors for DS IV are derivatives therefrom. The flanks for the DS IV shuttle plasmid were obtained using PCR on viral genomic DNA. Insertion into the backbone of the DS III shuttle vector was carried out with restriction sites PciI, NheI, NotI, or DraIII, which were contained in the primers (all restriction enzymes were from New England Biolabs, Frankfurt am Main, Germany). The integrity of all PCR-amplified regions was confirmed using Sanger sequencing on the final plasmids. The monofluorescent shuttle vector for insertion of mCherry into DS IV (p4PT-RED) was obtained using deletion/religation of a PmlI-PmlI fragment encoding EGFP, and for EGFP (p45T-GFP) through the deletion/religation of a HpaI/Klenow treated-NotI fragment encoding mCherry. The cloning and screening primers, together with expected amplicons, are shown in Table S1. PCR for this and all other PCRs described here were performed in a final volume of 25 µL with 0.15 µL Taq polymerase (Qiagen GmbH, Hilden, Germany), 200 nmol/L for each primer, and 125 µmol/L each nucleotide. Thermocycling was initiated at 94 °C for 80 s, followed by 35 cycles of 94 °C for 20 s, 55 °C for 20 s, 72 °C for 90 s, and terminated at 72 °C for 5 min. Primer sequences are listed in Table S1.

The tetherin-expressing shuttle plasmid was designed with the sequence Genbank XM_016157800 from the Rousette fruit bat (*Rousettus aegyptiacus*, Ra). The gene was optimised for human codon frequency and against poxviral sequences that may interfere with transcription [23,24]. A synthetic DNA (GeneArt/ThermoFisher Scientific, Schwerte, Germany) of the optimised tetherin sequence was inserted into the shuttle vector for DS IV as a BamHI/SwaI fragment that replaces EGFP in the DS IV dual shuttle plasmid, maintaining the strong mH5 promoter for tetherin.

After many attempts failed to generate recombinant viruses with this plasmid, expression of the tetherin selection was placed under the control of tetO-operator sequences. Tandem tetO elements were inserted immediately downstream of the promoter in such a way that Tet-repressor (TetR) proteins provided in trans can bind to the tetO sequences and interfere with transcription [25,26]. Different promoters were tested for their ability to suppress tetherin expression and were usually transferred as SpeI to BamHI fragments out of synthetic DNA into the same sites of the shuttle vector for DS IV. The shuttle vectors for most of the experiments described here express the tetherins from the hybrid promoter [27] followed by a tetO tandem repeat starting 2 bp downstream (named HYBdx promoter in this paper). The mCherry reporter protein as a visual control was provided in an antiparallel direction to tetherin and is expressed from the P11 promoter.

### 2.2. Generation and Testing of Recombinant MVAs

Recombinant viruses were obtained as described previously [10] through the infection of 1 × 10^6^ adherent CR.pIX cells in 6-well plates followed by transfection with shuttle plasmid, 90 min after infection. The infected/transfected culture (passage 0, P0) was lysed after 48 h and an aliquot was transferred to a fresh CR.pIX culture. Medium containing 1% methylcellulose, to interfere with virus diffusion, was applied 2 h post-infection with the P0 lysate. Foci were aspirated into 20 µL volumes after 48–72 h and transferred to 500 µL of medium. This suspension was sonicated for subsequent focus purification rounds and added to fresh cultures in 6-well plates, typically at 5 to 10-fold dilution. Again, a semisolid medium was added to prevent the diffusion and mixing of viruses.

Tetherin-expressing MVAs (tMVAs) were generated with the modification that adherent tCR9 cells (see below) were used for all steps. Focus isolation and purification were guided by the expression of fluorescent proteins. Fully focus-purified viruses were amplified once on adherent cells in 6-well plates prior to the production of larger stocks in suspension cultures as described previously [11]. The successful insertion, genome integrity, and purity of the obtained recombinants were verified using PCR analysis. For analysis of foci, total DNA was recovered from a 20 µL aliquot of the lysate of a purification step by adding 5 µL of QuickExtract™ DNA Extraction Solution (Cambio, Cambridge, UK) and heating to 65 °C for 10 min and 98 °C for 5 min. Five µL therefrom was used in a PCR reaction with a final volume of 25 µL. For a more detailed analysis that required a larger amount of DNA for several PCR reactions, genomic DNA was isolated with the Qiagen Blood purification kit according to the manufacturer’s instructions. Rapid recombination without focus picking is described in the results section.

### 2.3. CR.pIX and tCR9 Cell Lines

Continuous adherent and suspension cultures of CR.pIX cells from the muscovy duck were used for the experiments [28]. A cell line that stably expresses the TetR protein was used to suppress tetherin expression under the control of tetO-switchable promoters. This cell line, tCR9, was generated out of adherent and suspension CR.pIX cells, respectively, through the stable transfection of a linearised expression plasmid for tetR. The coding sequence in tetR was adjusted for the human codon frequency and (because vaccinia viruses replicate in the cytoplasm) did not contain a nuclear localisation sequence as opposed to commonly used TetR systems. The tetR open reading frame (ORF) was linked via an internal ribosome entry sequence to the ORF for puromycin. The expression of the mRNA is under the control of the human CMV promoter and was maintained by selection with 1 µg/mL puromycin (ThermoFisher Scientific, Schwerte, Germany).

Adherent CR.pIX and tCR9 cells were cultivated with DMEM:F12 (Gibco/Life Technologies, Carlsbad, CA, USA) containing 5% bovine serum (γ-irradiated, Australian-origin, Gibco). Suspension CR.pIX and tCR9 cells were cultivated with a CD-U7 chemically defined medium (Xell) in a shaking incubator (HT Multitron Cell, Infors AG, Bottmingen, Switzerland, Switzerland). The cultivation of cells, production of MVA in suspension by induction of aggregates, and sonication to obtain infectious units were carried out as described previously [10,29].

### 2.4. Quantification of Viruses by Titration and Expression of Fluorescence Markers

Infectious units were determined through titration on adherent CR.pIX cells in 96-well plates in replicates of 6 and serial dilutions of 10. Cytopathic effects are fully recognizable in CR.pIX cells even after infection at high dilutions (low MOI) 48 to 72 h after inoculation. The calculation to obtain TCID50/mL was performed according to the Spearman and Kärber method [30]. Viruses that encode tetherin were titrated on tCR9 cells.

Following recombination (passage 0, P0), lysates were used at appropriate dilution for obtaining separated foci that allow quantification to infect fresh layers of cells. A semisolid medium was added to the infected monolayer to interfere with diffusion, especially of MVA-CR19-derived infectious units, and to obtain precise foci for quantification. The cultures were scanned with the NyOne Scientific (SYNENTEC GmbH, Elmshorn, Germany) device and fluorescent foci were counted using the experimental NyOne Fluorescent Plaque Morphology (2F) (v. 0.9) module. The software module returns the number and area (in mm^2^) of fluorescent foci for each channel (here, green and red) and the total fluorescent area. Yellow (mixed channels) foci were calculated by first dividing the fluorescent area for each channel by the number of foci for that channel to obtain the mean area (size) of a focus for that channel. Next, the combined area of fluorescence for both channels was divided by the mean area of foci of one channel. This returns the number of foci of colour (green or red) that contribute to the combined (yellow) foci.

## 3. Results

### 3.1. Occupancy of DS IV Does Not Interfere with Viability of MVA-CR19

A large terminal duplication in MVA-CR19 has placed DS IV in the centre of the repeat region [10] (Figure 1a). The terminal repeats function as the origin of replication for genome amplification [13] and large changes to these structures can render a virus unstable [31]. We, therefore, asked whether viable viruses can be obtained after recombination of a reporter gene into DS IV of MVA-CR19 that would at least transiently reduce the extent of the terminal homologies, and (by performing the recombination with a mix of shuttle plasmids) whether different reporter genes can be inserted into the two distant locations (Figure 1b).

The two shuttle plasmids (with expression cassettes for EGFP or mCherry, respectively) were added at a 1:1 ratio for recombination with either MVA WT or CR19. Following recombination (passage 0, P0), fresh layers of cells were infected with lysates at appropriate dilution for obtaining separate foci and cultivated under a semisolid medium to inhibit the diffusion of viral particles for 48 h. The cultures were scanned and quantified with the NyOne Scientific (Synentec) device. As expected, most foci of the first passage were caused by infection with the parental virus and thus were without reporter-gene expression. Foci that expressed reporter usually were mono-fluorescent red or green. Yellow foci were found in recombination experiments with both WT and CR19. As there is only one DS IV in WT viruses, dual fluorescence is consistent with co-infection of viruses that express either GFP or mCherry. Another indication of co-infection, even at higher lysate dilutions, is foci that appear yellow at low magnification but show non-overlapping red and green fluorescence at higher magnification (Figure S1).

**Figure 2:**
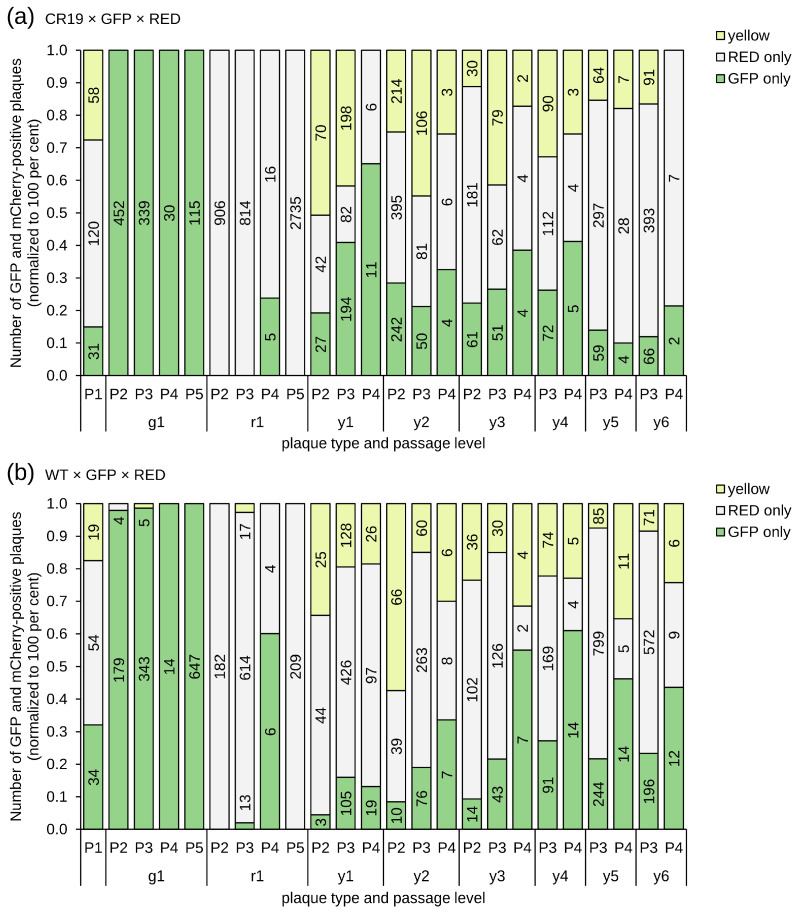
Relative composition of focus populations isolated after simultaneous recombination of MVA-CR19 (**a**) and WT virus (**b**) with shuttle plasmids for EGFP and mCherry reporters. The numbers in the columns give absolute focus numbers. Shown here is the development of one green (g1), red (r1) and six independently picked yellow (y1–y6) foci. Red and green foci could be purified, and yellow foci were always dispersed into a mixture of red and green foci in addition to yellow foci.

From both experimental threads red, green, and yellow foci were isolated for the infection of fresh monolayers in six-well plates (P2). This process was repeated three times (obtaining P3, P4, and P5). The number of foci at each passaging step was quantified by automated counting with the NyOne Scientific (Synentec) cell imager. As shown in Figure 2, pure monofluorescent foci, either red or green, were obtained within five passages for both WT and strain CR19 viruses. However, all yellow foci for both WT and strain CR19 separated into green and red foci (in addition to yellow foci) in subsequent passages. The observation that fluorescent foci can be passaged suggests that the insertion of recombinant sequences into DS IV is possible in MVA-CR19, and thus into a region that is duplicated at both termini of the genome and possibly important for replication. However, repeated separation of yellow into monofluorescent foci suggests that different recombinant sequences may not be tolerated in the ITRs.

### 3.2. Consecutive Insertion of EGFP into mCherry-Viruses: An Unusual Recombination Event

To characterise the insertions into the duplicated sites in the ITRs in greater detail, monofluorescent viruses, both WT and CR19, with reporters in DS IV, were isolated for additional experiments. PCR over the complete insertion site confirms the presence of EGFP or mCherry in DS IV. That no signal for native DS IV is observed for CR19 viruses suggests that both sites are occupied (Figure S2). Viruses were named according to the virus backbone and recombined shuttle plasmid (MVA-WT.4PT-RED, MVA-WT.45T-GFP, MVA-CR19.4PT-RED and MVA-CR19.45T-GFP) and were tested for purity 48 h post-infection through an analysis of fluorescence distribution (Figure 2, results for P5) and PCR analysis (Figure S2). As summarised in Figure 1, the presence of DS I allows for the differentiation of WT and CR19. Clear signals for DS I were visible with the genomic DNA of WT viruses, and no signal for DS I was visible when using the genomic DNA of CR19 as a template. PCR with primers against DS VI served as an internal control and gave the expected amplicon. Consistent with the monofluorescence phenotype, an occupancy with either mCherry or EGFP in DS IV was confirmed. Importantly, no indications of an empty DS IV (either because of contamination with parental virus or due to later deletions) were detected. A full lysate was generated for each virus to continue a study based on consecutive insertion of genes of interest into the ITR.

In the first set of experiments, the shuttle plasmid for EGFP (p45T-GFP) was recombined into wild-type and MVA-CR19 viruses, which already contain mCherry in DS IV (MVA-A3.4PT-RED and MVA-CR19.4PT-RED). Given that DS IV is duplicated in MVA-CR19, three potential outcomes can be anticipated: The replacement of mCherry at both telomeres using EGFP results in a green phenotype and G-G genotype. No changes for mCherry at both termini maintain the red focus phenotype and R-R genotype. Alternatively, the insertion of GFP at one telomere while mCherry is maintained at the other produces a focus with mixed colours, here termed yellow. The corresponding genotype is represented by R-G or G-R. If the two telomeres are aligned during replication, then a conversion is to be expected from yellow to a single colour (red or green, not both; from R-G/G-R to R-R or G-G) (Figure 1b).

**Figure 3:**
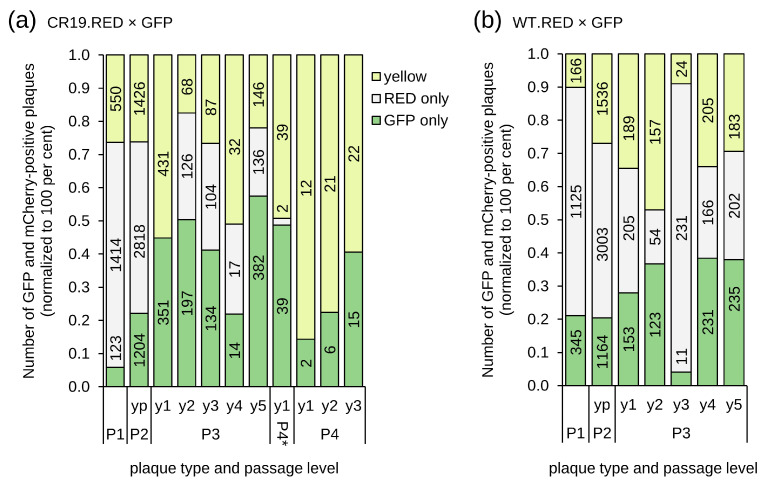
Phenotype distribution of foci after recombination of WT (**a**) and CR19 viruses (**b**) that already carry mCherry in DS IV with a shuttle plasmid that codes for EGFP. Yellow foci were picked into pools (py) at passages 1 and 2. The foci were not pooled at passages 3–5 (individually named y1–y5). For WT, all yellow pools and yellow clones were separated into green, red, and yellow components independent of passage level. For CR19, a clone was identified in P3 (y1) that had no apparent red components and a very high frequency of yellow. That clone was focus-picked for a further 2 passages (P4 and P5, P4* is an independent passage of the same clone).

Virus diffusion was again inhibited by the addition of a semisolid medium, and each passage was quantified using automated NyOne focus counting (Figure 3). After 2 days, 7 WT-derived and 38 CR19-derived yellow P1 foci were picked out of a background of predominately parental red foci (parental viruses, 85% of the 1660 P1 foci for CR19 and 69% of the 1636 foci for WT) and green foci (newly formed recombinants, 7% for CR19 and 21% for WT). The yellow foci were pooled and diluted for infection of fresh monolayers of CR.pIX cells in six-well plates. The results of the automated counting returned a total of 5448 P2 foci for CR19 and 5703 for WT. The majority of the obtained foci are monofluorescent red or green (74% for CR19 and 73% for WT). Although yellow foci were picked for subsequent analysis, only 26% of the foci with CR19 background and 27% with CR19 background were again yellow in passage 2. The similar frequencies of fluorescence phenotypes for WT and CR19 (viruses with a single versus two DS IV loci) can be most plausibly explained by assuming that, in both experiments, the isolated yellow foci were caused by co-infection with different fluorescence genotypes.

Five yellow foci of both WT and strain CR19 were picked for subsequent individual passaging under methylcellulose without pooling. The expression patterns of the foci that appeared to be yellow in the preceding passage were again comparable for both WT and CR19: most infections had similar numbers of red and green foci, with the proportion of yellow foci usually in a range of less than 50% (Figure 3). There were no non-fluorescent foci.

**Figure 4:**
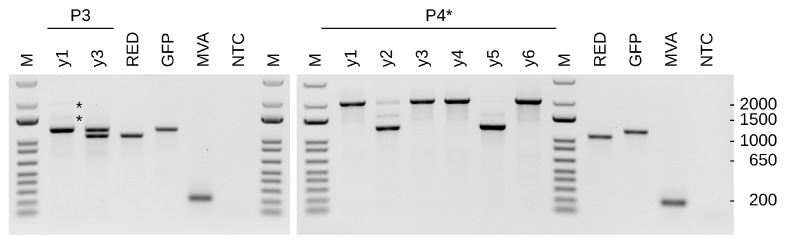
Insertion at DS IV has increased in size for a consistently yellow focus. Recombination may have occurred by a chance integration of different inserts at the distal termini. Intermediate bands that may be caused by transitory states are weakly visible in P3 y1 and are marked with asterisks (*). The foci shown in the right panel are foci from P4* (that were derived from P3 y1) in Figure 3. RED and GFP for reference PCRs with shuttle plasmids as a template, NTC for non-template control, and MVA represents a wild-type virus without insertion in DS IV. The size marker in this and all other agarose gels is the 1 kb Plus DNA Ladder (ThermoFisher).

Only for one isolate (P3 y1 of CR19) did infection result in a greater number of yellow than green and the absence of red signals. This isolate was further investigated through passaging (P4 and P5, Figure 3a) and PCR (P4 * foci y1-y6, Figure 4). The continued presence of predominantly yellow and mono-fluorescent green foci under methylcellulose suggests that true dual (red and green) reporter expression has been established. The observation that red fluorescence was reduced compared to previous expression levels, and the green fluorescence is consistent with possible promoter interference or a genetic defect in the P11 promoter. Non-fluorescence (reversion to ancestral genotype) was not detected.

DS IV PCR primers (Table S1) were chosen to amplify the complete insertion cassette including portions of the recombination flanks so that potential contamination by WT and occurrences of partial deletions can be observed. The MVA-CR19 isolate P3 y1 gave a major amplification product at the same height as the p45T-GFP shuttle plasmid control and very faint signals for 1.5 kb and 2 kb inserts. The unexpectedly large 2 kb amplicon was observed as the major amplification product for four of the subsequent P4* isolates (Figure 4). Sequencing of this fragment confirmed the presence of a large fragment in DS IV that is capable of expressing both reporters.

Two isolates showed the expected 1099 bp for the 4PT-RED cassette as the dominant band and, again, weak signals at 1.5 kb and 2 kb. The 1.5 kb middle band may represent a recombination intermediate. We excised this band; however, sequencing returned chromatograms with too much noise for interpretation.

A six-well plate was inoculated with a diluted lysate of the P3 y1 isolate. From this P4 plate, foci were picked and used to obtain an additional passage (P5). The P4 and P5 plates were analysed 2 days post-infection and maintained a majority of yellow foci without the presence of red foci (Figure 3a). A combined expression cassette for both reporters from DS IV was furthermore confirmed by PCR (Figure S3) for 7/7 isolated foci of P4 y1 and 6/7 isolated foci of P4 y2. None of the tested isolates in any of the passages gave amplicons indicative of reversion to an empty DS IV.

### 3.3. mCherry into EGFP-Expressing MVA-CR19: Confirmation of Copy-Correction at the Terminal Positions

**Figure 5:**
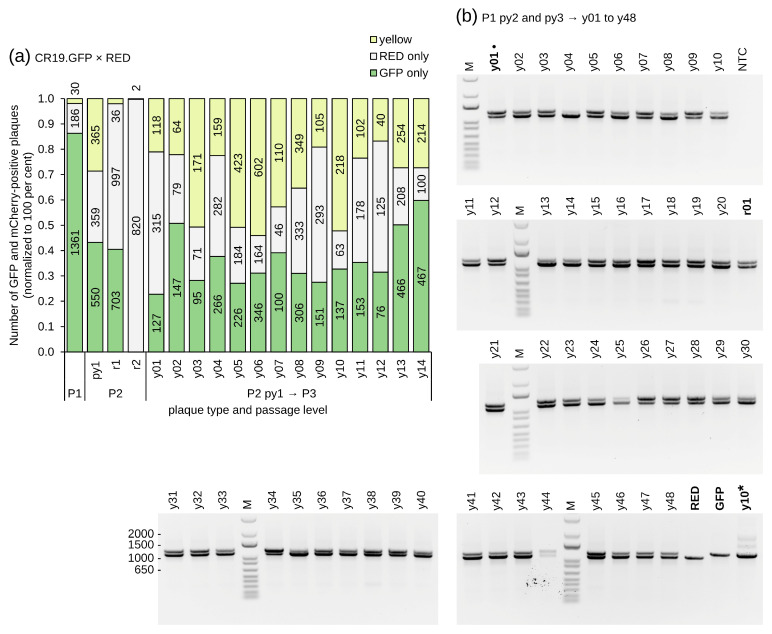
Quantification of foci that were obtained in a replacement of an EGFP expression cassette in DS IV of MVA-CR19 against mCherry expression cassette: (**a**) Foci with a predominantly monofluorescent red phenotype were obtained after 2 passages. A pure yellow phenotype was not recovered and a high frequency of distinct red or green foci was observed for all 14 isolated yellow P3 foci. (**b**) Confirmation that recombination towards large dual-expressing insert is a rare event. Focus y01^•^ was also compared to a pure yellow focus in a comet assay, see Figure S5. PCR with r01 is a positive control for PCR on known viral DNA with the mixed isolates in Figure S4 and the partially consistent yellow isolate y10 in Figure S3 (here denoted as y10*). RED and GFP are reference controls as described above. Expected amplicons are 1099 bp for DS IV containing mCherry, 1231 bp for DS IV containing EGFP, approx. 2000 bp for a combination of both reporter genes, and 220 bp for an empty (WT) DS IV (see also Supplementary Table S1).

To supplement the preceding two experiments with earlier time points for the PCR and to address the formal possibility that the pre-existing structure of the (mCherry) insert in DS IV may influence the outcome, we also performed recombination of the mCherry shuttle plasmid into an already GFP-containing virus. Infection and transfection were performed in a well of a six-well plate as described above. A lysate was prepared after 2 days of the infected/transfected reaction and applied to fresh cell monolayers in a six-well plate at dilutions from 500-fold to million-fold. This P1 plate was scanned after 3 days, and 7 red-only and 36 yellow foci were picked (out of a total of 1577 obtained foci, Figure 5a first (P1) column).

Red-only P1 foci (Figure 5, first column) obtained with a previously green virus can be formed by complete insert replacements on both termini or, less likely, through the insertion of mCherry into one and deletion of EGFP at another telomere. In a test using PCR against DS IV (Figure S4), there were no empty DS IV signals in seven isolates, which is consistent with a complete replacement within a single replication cycle.

We also investigated whether the unusual recombination could be observed again where only one homologous flank contributes to a larger dual expression cassette. Fifteen P1 foci with phenotype for an R-G or G-R genotype (that is, green and red fluorescence with similar intensities and good overlap of fluorescent areas) were selected and five foci each were pooled (obtaining P1 pools py1–py3).

Pool P1 py1 was used to infect a six-well plate. Similar numbers of green, red, and yellow P2 foci were observed. Fourteen P2 yellow foci were picked, and all gave mixed populations of green, red, and yellow foci in P3, suggesting that the genotype capable of expressing both EGFP and mCherry via a single infection has not been obtained (Figure 5a).

The other two pools (P1 py2 and py3) were used to inoculate a fresh cell monolayer. Most of the foci were mono-fluorescent red or green. Forty-eight yellow of the P2 foci were isolated and tested using PCR against DS IV. Consistent with the previous mixed fluorescence phenotypes due to co-infection, all PCR reactions resulted in a double band typical for separate GFP and mCherry cassettes (Figure 5b), and none produced a band that suggested either an empty DS IV or dual-reporter recombination.

In summary, recombinant viruses that contain reporter genes at distant corresponding sites, situated almost in the centre of each of the two terminal repeats, can be generated and stably passaged. The occurrence of foci with mixed colours (green and red) was infrequent in the course of our experiments. The appearance of dual fluorescence was shown at higher magnification to be caused by the superimposed foci of different colours (Figure S1). PCR reactions with viruses purified out of these foci usually separated into either R-R or G-G viruses in subsequent passages. Possibly, a very rare exception of true dual fluorescence was observed. However, these viruses did not show two distinct mCherry and EGFP amplicons in the separate DS IV sites but rather a novel larger amplicon indicative of recombination towards an RG-GR genotype. PCR in the passages immediately after recombination suggested that in the mixed populations of green and red foci, conversion to a single fluorescence occurs rapidly and appears to be stable, as no deletions towards empty DS IV were encountered.

### 3.4. Insertion of a Selection Marker

To investigate whether genes for therapeutic proteins or vaccine antigens that may impede viral replication [26] could also be inserted into the telomer, we designed experiments with a selection marker to accelerate the generation of recombinant viral vectors. The initial aim was to utilise the property of MVA-CR19 to release higher amounts of infectious particles into the culture supernatant [11,32]. The rationale was to introduce a potential selection marker into DS IV that would restrict the release of the infectious units. An exchange of the selection marker against the gene of interest would reestablish the CR19 phenotype so that recombinant viruses (that have lost the selection marker) would be enriched in the culture supernatant. One protein that may be suitable for interfering with the diffusion of infectious particles is a factor of the innate immune system, tetherin [33], and such a protein may, therefore, also be a good candidate to test the limits of tolerance to expression in a recombinant MVA telomere. To compare effects, we attempted to insert an expression cassette for tetherin from the rousette bat into WT and CR19 MVA.

mCherry under the control of the late P11 promoter was co-expressed from an antiparallel position directly next to tetherin to facilitate identification of recombinant viruses. However, multiple attempts failed to yield viruses (WT or CR19) that contained such a dual expression construct. We, therefore, decided to generate a derivative of the CR.pIX cell line (called tCR9) that constitutively expresses TetR without a nuclear localisation signal and to put tetherin in the viral genome under the control of an early/late hybrid promoter [27] or the strong mH5 promoter [34] followed by tetO sequences (called HYBdx and M52dx, respectively). MVA coding for tetherin with such a design does not express tetherin unless doxycycline is added to release the block of TetR on transcription [35]. With such a setup, we were able to obtain recombinant viruses that contain the tetherin/mCherry dual expression cassette in the DS IV of WT and CR19, respectively.

### 3.5. Tetherin Active Also If Activated Late in Replication

**Figure 6:**
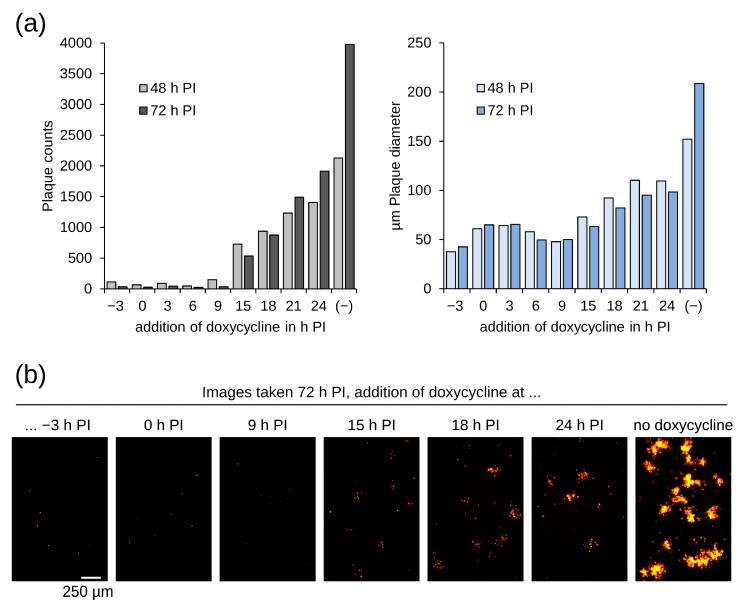
The addition of doxycycline has clear effects on the replication of tetherin-inducible viruses even if added 24 h post-infection. (**a**) Infection was performed with an MOI of 0.01 in adherent cultures. The tested virus is MVA-CR19 with tetherin in DS IV under the control of the HYBdx promoter. Co-expression of mCherry is used to quantify replication. Both, the number (left panel) and size of foci (right panel) are reduced compared to the uninhibited control, symbolised by (−). (**b**) The appearance of infected cultures that are quantified in (**a**) after doxycycline-induced de-blocking of tetherin expression.

Vaccinia virus genome replication typically peaks between 3 h and 4 h post-infection [36]. Virus recombination is first detected between 5 h and 6 h post-infection, the same time window the assembly of mature virions occurs [36]. Morphogenesis towards wrapped virions and subsequent release of a fraction thereof as extracellular enveloped virions is most active 8 h to 16 h post-infection; however, the first egress can be detected as early as 4–6 h post-infection [36,37]. To test the stringency of tetherin interference with replication, doxycycline was added at sequentially later time points after infection (Figure 6). The impact of tetherin expression was visible at the level of focus numbers and fluorescent focus sizes even if the promoter for the marker was de-blocked as late as 24 h post-infection.

**Figure 7:**
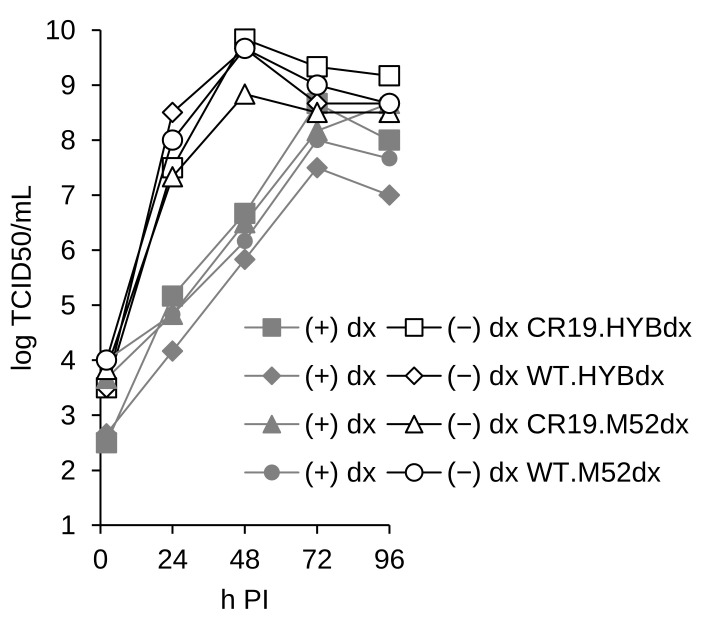
Infectious titers of tetherin-containing viruses in suspension cultures. WT and MVA-CR19 viruses replicate to high titers if the expression of tetherin is suppressed (open symbols) and to 1000-fold lower titers if the expression is deblocked. MVA-CR19 contains two copies of tetherin and replicates to lower titers also if tetherin is under the control of the strong M52dx promoter. This experiment was performed twice with similar results.

The effect on plaque counts in adherent cultures was confirmed for infectious titers in replication kinetics (Figure 7). For this experiment, suspension tCR9 cells were infected with different viruses at an MOI of 0.05 in the presence or absence of doxycycline. We compared the replication of MVA-CR19 and WT with tetherin in DS IV under the control of HYBdx or mH52dx, respectively. While the replication curve in the absence of the inducer is similar for the viruses (with one exception, open symbols), titers are three orders of magnitudes lower than 48 h post-infection if tetherin expression is allowed (bold symbols). The exception in the replication kinetics in the absence of the inducer is the CR19-derived virus that expresses tetherin from the mH5dx-promoter. We hypothesise that the strong mH5 promoter exhibits leaky expression also in the repressed state. As a consequence, CR19-derived viruses (but not corresponding WT viruses) are closer to a threshold, where tetherin background expression interferes with replication because of the higher gene doses due to the duplicated DS IV sites.

### 3.6. Marker Replacement

We next tested whether the observed strong interference of tetherin with virus replication can be utilised to obtain pure recombinant viruses faster by marker-assisted replacement with a gene of interest. Monolayers of tCR9 cells in six-well plates were infected with the CR19-derived virus that carries the expression cassette for tetherin in DS IV under the control of the hybrid promoter. A shuttle plasmid was transfected that contained mCherry in the same orientation as mCherry in the virus and EGFP in the same orientation as tetherin. With such a setup, successful replacement of tetherin yields green fluorescence in the desired recombinants and correct homologous recombination maintains red fluorescence. The experimental steps for the generation of recombinant viruses based on tetherin selection are shown schematically in Figure S6.

**Figure 8:**
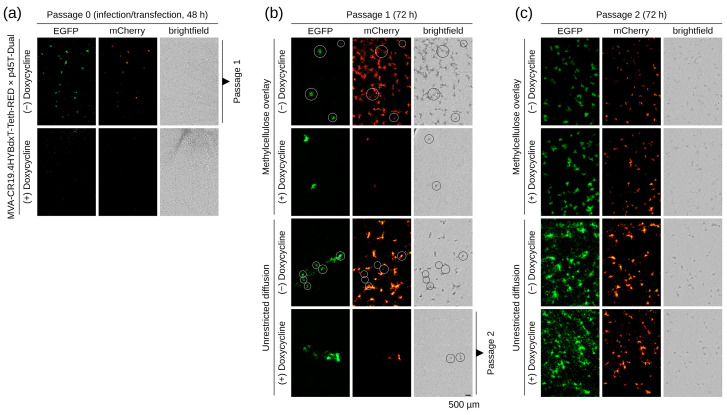
The tetherin expression cassette is replaced within one passage. Successful replacement of the tetherin-containing insertion in DS IV through the dual-expression cassette in the shuttle plasmid for the same DS IV results in a loss of tetherin expression and gain of EGFP expression. Faithful homologous recombination furthermore maintains the constitutive expression of mCherry: (**a**) Infection and transfection for recombination without application of the semisolid medium. Only if tetherin expression is blocked virus can replicate for successful recombination. (**b**) A lysate of the infected/transfected cultures was transferred to a fresh cell monolayer in the presence or absence of doxycycline. A semisolid medium was applied for quantification. No parental viruses appear to be present if tetherin expression is allowed. (**c**) A lysate of the doxycycline-treated well of passage 2 was used to infect a fresh cell monolayer. All observed viruses appear to be desired recombinants, independent of further induction with doxycycline.

As expected, when doxycycline was added at the time of recombination to de-repress tetherin expression, no replication of the receiving viruses was observed (Figure 8a). EGFP from the transected shuttle plasmid was visible at very low frequencies, and no recombinants were obtained in the second passage. If tetherin expression was kept suppressed during infection/transfection then EGFP expression (from the viral promoter in the shuttle plasmid) overlapped consistently with mCherry expression. A lysate of this culture (10–30 µL of the 2 mL lysate of a well of a six-well plate) was transferred to fresh tCR9 monolayers in a 12-well plate with or without semisolid medium and/or doxycycline (Figure 8b). The addition of a semisolid medium facilitates the identification of foci and in the absence of doxycycline shows a large background of red events that are not green, indicative of infection with the parental virus. The addition of doxycycline activates tetherin expression so that only EGFP-positive foci are visible in the treated wells. Parental viruses were not detected.

Without focus-picking, a lysate was prepared of the passage 1 culture without semisolid medium but under doxycycline for selection against parental virus. Aliquots of this lysate were added to fresh monolayers of tCR9 cells (Figure 8c). At this stage, there were no obvious differences in replication in the presence or absence of doxycycline. All foci appeared to be positive for both fluorescence markers, and there were no mono-fluorescent foci and no non-fluorescent foci.

The automated quantification with the plate reader gave a 27-fold excess of parental (monofluorescent red foci) in the first passage under a semisolid medium without doxycycline (Figure S7). With doxycycline, the ratio was only 0.7-fold. The ratios of green to red foci were similar for the infected cultures at passage 2 and close to the expected parity of 1.0 when all recombinants have exchanged tetherin against EGFP and maintain mCherry, 1.2-fold (without) and 1.1-fold (with doxycycline).

**Figure 9:**
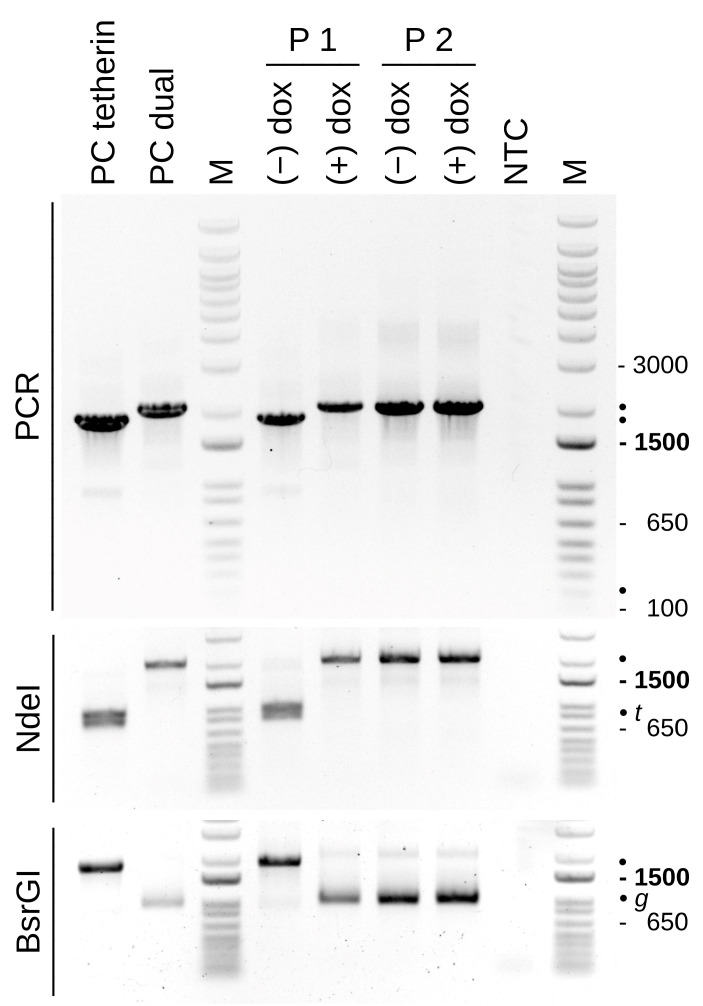
PCR over DS IV confirms the successful replacement of the tetherin marker with the dual expression cassette within a single passage. The upper panel shows the PCR reaction. The amplicons for tetherin/mCherry and EGFP/mCherry cassettes are similar in size (1839 bp and 2016 bp, respectively, marked by the bullets on the right side). Restriction enzyme digestion was employed to confirm the identity of the amplicons, NdeI within the tetherin gene (resulting in 986 bp and 853 bp, expected position indicated with the *t*) and BsrGI within EGFP (leaving 1025 bp and 991 bp, indicated with *g*). Left two lanes to show PCR on the shuttle plasmids as a positive control (PC). NTC for non-template control. An empty DS IV would give a signal of 220 bp.

The phenotypic results are confirmed in a PCR reaction with genomic DNA isolated at passages 1 and 2 in the presence or absence of doxycycline with the tetherin and dual shuttle plasmids as positive controls (Figure 9). For passage 1 populations cultivated without doxycycline, only viruses with tetherin and no amplicons containing the dual cassette were detected, whereas, in the presence of doxycycline, no tetherin-containing viruses appear to have replicated. With the second passage out of the doxycycline/tetherin-selected cultivation, only the desired recombinants that contained the dual expression cassette emerged. The PCR over the complete insertion site did not suggest the presence of irregular recombinations such as partial or complete deletions in DS IV in both passages, independent of cultivation with or without doxycycline. Because the amplicons for the parental and intended recombinants are similar in size (1839 bp and 2064 bp, respectively) restriction fragment length polymorphism was used to further confirm the results. In summary, recombinant viruses free of the parental virus were obtained without focus-picking within two passages after infection/transfection by using tetherin as a selection marker.

## 4. Discussion

The inverted terminal repeats (ITRs) of poxviruses are stretches of homology that flank the genomic core region and can be less than 1 kb [38] to more than 20 kb [10,17] long. They are covalently sealed so that no 3′ or 5′ ends are exposed and terminal hairpin loops are formed (reviewed by [12]). Multiplication of the poxviral genome is not yet fully understood due to the complexity of autonomous replication of genomic DNA in the cytoplasm and the lack of in vitro replication systems [39]. In analogy to the similar organisation of the terminal repeats of parvoviruses, poxvirus replication is proposed to be most likely initiated within the apical hairpin structures (that are not perfect helices but contain unpaired bases) [12]. Synthesis appears to be asymmetrical by a rolling hairpin model, involving a leading strand and possibly bursts of discontinuous Okazaki fragments [12,13,39]. Concatemers of full-length genomic segments are formed and predicted to be separated by Holliday junctions, which are subsequently resolved into unit genomes by a viral endonuclease [16]. It is possible that, during this step, the two distant ITRs are aligned in such a way that identical sequences are restored at both ends, with circular replication intermediates being one mechanism first proposed in 1979 [40].

Recombinant poxviruses are typically obtained through homologous recombination with appropriately designed plasmid DNA (the shuttle plasmid) that is transfected into cells shortly after infection [41,42,43]. The recombination flanks in our shuttle plasmid were complementary to DS IV, one of the markers that MVA acquired during the successive attenuating passaging [4,5,9]. The loss of sequences leading to DS IV has disrupted MVA188R (variola virus gene B22R), which is still in the core of WT MVA, 1078 bp upstream of the right ITR [5,44]. The rearrangement of ITRs in MVA-CR19 [10] has occurred at 16,386 bp upstream of DS IV; therefore, this site is now duplicated at both termini, 10,722 bp from the hairpin loop/apex and 16,401 bp from the core, respectively.

The first set of experiments has revealed that simultaneous recombination for the insertion of EGFP or mCherry into the ITRs of MVA-CR19 does not interfere with replication but surprisingly has also resulted in CR19 viruses that were consistently positive for only one fluorescent reporter. To test more rigorously whether different genes are tolerated in the two ITRs, monofluorescent viruses from the first round were used for recombination with the corresponding other reporter (“green” viruses recombined with a shuttle plasmid for mCherry and “red” viruses for EGFP). Again, with the exception of an unusual recombination, yellow foci were caused by mixed infections. The observed unusual recombination resulted in identical large expression cassettes for both reporters in both termini. This probably occurred during the initial reaction with the shuttle plasmid and was perpetuated during replication. Non-homologous recombination may be caused if strand breaks at the replication fork are accidentally repaired by priming with unrelated DNA and is therefore expected to be a rare event [45]. Consistent with the literature, with the exception of this single occurrence, we did not observe signs of non-homologous recombination events in a subsequent screen of recombinant genotypes at passage levels 1 and 2, although we focused on the investigation of mixed double infections by selecting yellow foci.

While the genome core contains genes essential for replication, the ITRs of different orthopoxvirus species can encode a variable number of accessory proteins that contribute to immune evasion and host range [44,46]. Transgene insertion into the ITR may also automatically duplicate the gene doses and may even be maintained with greater stability if deletions at one site are corrected through the re-insertion of the distant remaining copy. However, the ITRs are also known to expand and contract in adaptation to selective pressure and can accumulate significant heterogeneity [17,47,48]. We have, therefore, also tested whether a gene that interferes with replication can be maintained in the ITRs sufficiently long enough to establish a novel selection system.

The initial aim was to functionalised intended insertion sites with a marker that reduces the release of extracellular infectious units of MVA-CR19 to the low levels of the WT virus [11]. Replacement of that marker against a gene of interest would allow the harvest of the desired recombinant viruses directly from the supernatant. Among proteins that interfere with the mobility of extracellular infectious units are tetherins. These are components of the innate immune system with a transmembrane domain at one and a glycosylphosphatidylinositol (GPI) anchor at the other terminus [33]. The two lipid anchors are connected by a coiled coil that is also predicted to mediate the dimerisation of tetherin monomers. Tetherins appear to accumulate at sites of the plasma membrane where enveloped viruses complete the last step of morphogenesis. As the viruses bud through the membrane, tetherins remain anchored to the host cell but also become incorporated into the viral envelope and thus interfere with the release of particles. Because human tetherin may not be functional against poxviruses [49], we chose tetherin from a chiropteran. Bats are known for a robust innate immune response against various pathogens [50] and have also experienced a profound evolution of tetherins [51].

Vaccinia viruses spread efficiently via cell-to-cell contact [52], and we therefore expected that cell-free transmission of infectious units would be impaired by the expression of tetherin. However, recombinant viruses capable of expressing tetherin could only be generated with a switchable viral promoter for the selection marker. Tetherin visibly interfered with the multiplication of both WT and CR19 in adherent cell monolayers even if the expression was switched on as late as 24 h post-infection. In suspension cultures, the yields of infectious units were 1000-fold lower if tetherin was expressed.

The strong effect of tetherin may be explained by the property of poxviruses to bud into intracellular vesicles, either Golgi-derived cisternae or multivesicular bodies [53]. As opposed to viruses that bud from the cell surface, they may, therefore, encounter tetherins very early during morphogenesis, possibly already at the time when the viral factories are reorganised into the characteristic crescent-like structures for the assembly of the immature virions (IVs) [54,55,56,57].

Poxviruses, including MVA, are promising candidate vectors for immunotherapy against tumours [19,20,21]. However, the reported time from identification of a gene of interest to generation and release of a recombinant poxvirus ranges from 42 to 84 days, which is suboptimal for both pandemic preparedness [22] and personalised medicine for cancer treatment [20]. One challenge for obtaining MVA constructs is that very few recombinant viruses, only 0.1% of the population, are generated [42] that must be identified and isolated by experienced operators in successive rounds of infections. The identification of recombinant viruses for plaque purification can be facilitated with fluorescent markers such as EGFP, co-expressed with the introduced gene of interest. To remove any accessory sequences in a vector for clinical use, the marker is equipped on both sides with short, approximately 300 bp-long homologous flanks for intragenic deletion during subsequent passaging of candidate viruses. If the fluorescent marker is additionally fused to a host range factor, viruses with limited tropism can be further enriched by replication in a cell line that is not permissive for the parental viruses [58,59]. Related developments depend not on the addition but deletion of specific host range factors to obtain a parental virus with impaired replication properties. The shuttle plasmid is designed to contain the deleted host range gene together with the gene of interest so that the full replication potential is restored by the recombination [60]. However, both methods are only applicable to viruses (such as MVA) where the mechanism for a restricted host range compared to ancestral viruses is known and suitable trans-complementing factors are available. The plaques furthermore still need to be accessed visually. Further disadvantages of the described approaches are that the intragenic deletion of the marker requires several passages in the former protocol and that insertion can be performed only in the initial host range locus in the latter protocol.

Using tetherin as a marker for replacement with genes of interest appears to enrich the relative ratio of recombinants over parental viruses to allow the recovery of the desired constructs without focus picking and within two passages after in vitro recombination. Once functionalised according to conventional methods, the receiving tetherin-MVAs can be used for screening experiments with variants of antigens in the same genomic site or rapid conversion into personalised therapeutics.

Additional experiments are needed to address the limitations of our study. For example, it would be interesting to learn more about the dynamics of the recombination at the two ITRs. Tracking changes using PCR and the expression of fluorescing reporters provides an average of a population. Cas9-targeted direct sequencing of passage 0 populations (at the time of recombination) may help to elucidate the mechanistic steps of the insertion. For example, whether insertion into one ITR is favoured over the other (possibly the ITR where replication initiates), and where a newly functionalised ITR is located in genome concatemers relative to the parental ITRs. The length of poxviral ITRs varies widely, and it would also be interesting to test with increasingly longer insertions how a new boundary between core and ITR is defined so that the two ITRs are not aligned anymore.

With the current data, we can only speculate as to how tetherin may cause its strong effects. Electron microscopy may help to investigate differences in the maturation and migration of viral particles in the presence and absence of tetherin expression. From an application perspective, switchable promoters are often not completely silent; therefore, selective pressures for the deletion of tetherin in the functionalised viruses may exist during preparation for the actual replacement. As very few viruses are required for recombination, stability is not an overarching issue in this case: infection of a microtiter plate with an MOI of 0.01 corresponds to 10,000 infectious units, and a single-passage infection in 30 mL suspension would be sufficient for more than 10^5^ recombinations (calculating with a yield of 30 mL × 10^8^ PFU/mL). Still, we are assessing stability and additional promoter designs for these purposes.

## 5. Conclusions

To summarise, we have demonstrated that a site almost in the centre of the ITRs can be targeted by homologous recombination for the expression of transgenes. One advantage associated with such a site is the maintenance of identical cassettes and doubling of the gene doses. Foreign genetic sequences in the viral telomeres were sufficiently stable for the expression of a novel selection marker for vaccinia viruses, tetherin, which demonstrated the capacity to facilitate the rescue of recombinant vectors within two passages and without the need for focus picking procedures.

## 6. Patents

Patent applications with data from the work reported in this manuscript have been filed.

## Data Availability

The raw data supporting the conclusions of this article will be made available by the authors upon reasonable request.

## Supplementary Materials

The following supporting information can be downloaded at: https://www.mdpi.com/article/10.3390/vaccines12121377/s1, Figure S1: Co-infection complicates the identification of recombinant MVAs; Figure S2: MVA-CR19 tolerates recombinant inserts at the duplicated DS IV in the viral telomers; Figure S3: PCR on DS IV of viruses with a consistent yellow phenotype that appears to contain a large insertion at both DS IV sites; Figure S4: Recombination of mCherry into EGFP-containing MVA; Figure S5: Separation of GFP and mCherry signals in a mixed population but not in the clone containing the single large insert; Figure S6: Tetherin-assisted generation of recombinant MVA (rMVA); Figure S7: Automated counting of foci after tetherin-assisted recombination; Table S1: Primer sequences and expected amplicons.
