# Supplementary material for: Expression of an Efficient Selection Marker Out of a Duplicated Site in the ITRs of a Modified Vaccinia Virus Ankara (MVA)
Source: Vaccines (Basel). 2024 Dec 6;12(12):1377. doi: 10.3390/vaccines12121377 (PMC11680203; doi:10.3390/vaccines12121377)
Supplement: Supplementary file 1 [file vaccines-12-01377-s001.zip › vaccines-3337324-supplementary.pdf]

vaccines

Expression of an Efficient Selection Marker Out of a Duplicated Site in the ITRs of a Modified Vaccinia Virus Ankara (MVA)

Supplementary Material

Table S1. Primer sequences and expected amplicons.

Site	Sequence	Expected amplicons
DS IV left flank forward	ttacatgtgactgtgttcagggtatag	PciI-820 bp-NheI
DS IV left flank reverse	catagctagccgtacatccacatct	
DS IV right flank forward	ttagcggccgcgtacggctttagaaatgag	NotI-888 bp-DraIII
DS IV right flank reverse	ttcacgtagtgggtctgaactgggcac	
DS IV forward	gtgctataacgcgactatctag	MVA: 220 bp; dual: 2016 bp; GFP: 1231 bp; mCherry: 1099 bp; Ra tetherin: 1839 bp
DS IV reverse	tgttggtagttcttccgtgg	
DS V forward	cgtgtataacatctttgatagaatcag	MVA: 603 bp; tMVA-CR19: 2200 bp
DS V reverse	aacatagcggtgtactaattgattt	
DS VI forward	tttggtaatggtttctcatgtgg	MVA: 407 bp; tMVA-CR19: 2030 bp
DS VI reverse	gacatttagtttgagtgttcctg	
TK locus forward	ctctctagctaccaccgcaa	MVA: 286 bp; tMVA-CR19: 1946 bp
TK locus reverse	atgcgtccatagtcccgttc	
DS I forward	ctttcgcagcataagtagtatgtc	
DS I reverse	cattaccgcttcattcttatattc	

Co-infection complicates identification of recombinant MVAs

MVA causes co-infection also at lower MOIs and after sonication. Automated quantification of the foci by fluorescence may therefore overestimate yellow foci from separate infection events at the same position.

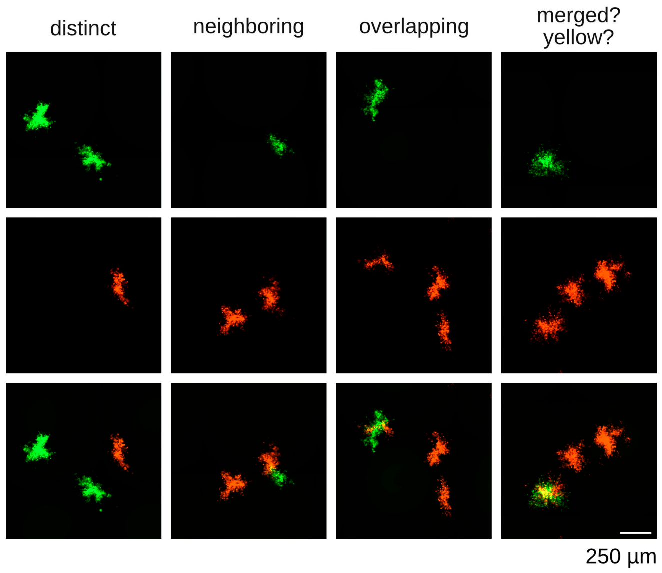

Figure S1. Foci that appeared to be yellow but are shown to be formed by different co-infecting viruses at higher magnification. Only the yellow and red foci in the right-most panel appear to be fully aligned.

The two telomeres of MVA-CR19 contain identical inserts

Transfection with two different shuttle plasmids for DS IV at the time of infection resulted in viruses that incorporated only one version of the recombinant DNA. The singly-positive viruses (at both sides of the genome in the case of MVA-CR19) were characterized by PCR to demonstrate homogenous insertion and non-empty insertion sites.

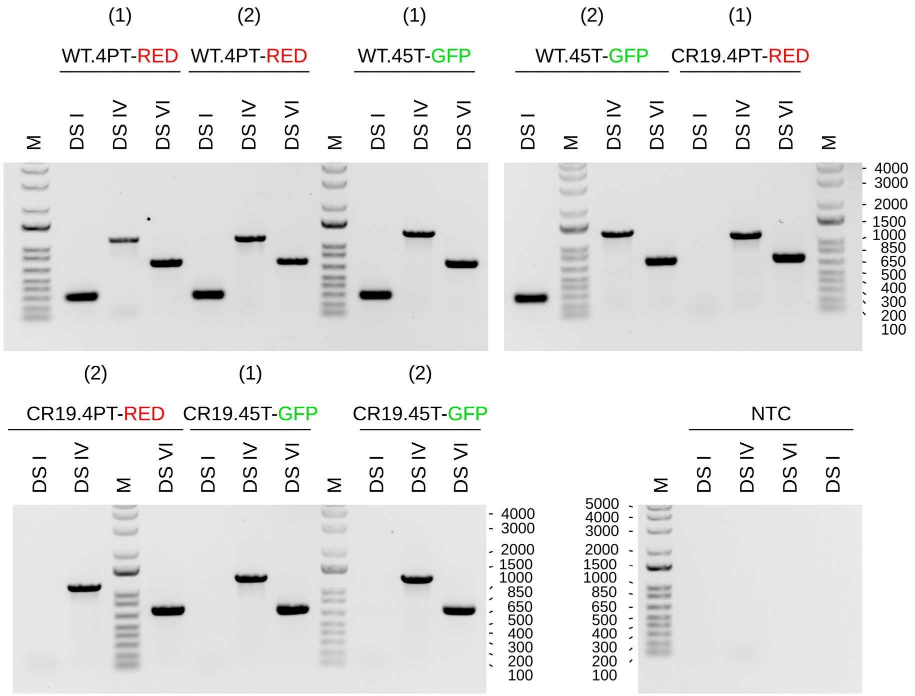

Figure S2. MVA-CR19 tolerates recombinant inserts at the duplicated DS IV in the viral telomers. Shown are results for two independently picked clones each of WT and MVA-CR19 with either EGFP or mCherry inserts, respectively. The expected amplicons are 291 bp for WT (empty) DS I, 1099 bp for 4PT-RED in DS IV, 1231 bp for 45T-GFP in DS IV, 220 bp for WT (empty) DS IV, and 702 bp for WT DS VI (see also Figure 1). CR19 has lost the DS I in the recombination that led to the duplication of DS IV. PCR non-template controls (NTC) were without signals and are on a separate gel. See Supplementary Table S1 for primer sequences.

Combined expression confirmed by PCR

Yellow foci typically split into red and green monofluorescent foci with each passage. One focus was identified (Figure 3 (a), passage 3 yellow 1 or P3 y1) that consistently gave a yellow fluorescence in successive passages. The genotype of this virus is consistent with a non-homologous recombination event that resulted in a large insertion at both termini capable of expression of both reporters.

Seven yellow foci each were isolated from P4 y1 (termed P5 y01-y07) and P4 y2 (P5 y08-y14). One green focus was picked of P4 y2 as a control (g01 in Figure S3). 13 of the 14 yellow isolates gave a single large amplification product without apparent variation among the different preparations. The single exception showed a dominant band at the level of the GFP signal, a minor band for the large (2 kb) amplification product and another minor band in between. This middle band may be an intermediate from monofluorescence towards the yellow phenotype or away from the yellow phenotype towards monofluorescence. We tried to isolate and sequence this middle band but could not obtain a sequence at suitable quality for interpretation. In a subsequent amplification of virus isolates with intermediate bands the PCR reactions gave only the smaller sized band for the 45T-GFP insert, supporting the hypothesis that the intermediate band is only transitory in a fast homology-directed copy pathway.

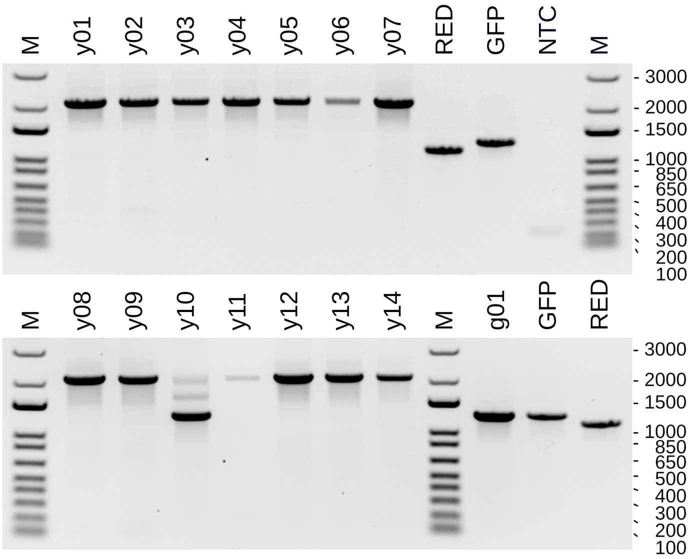

Figure S3. PCR on DS IV of viruses with a consistent yellow phenotype that appear to contain a large insertion at both DS IV sites. The large insertion appears to be stably maintained for 13 out of 14 independently picked clones at passage 5. RED and GFP for reference PCRs with shuttle plasmids as template, NTC for non-template control, g01 for a PCR on a virus that expresses EGFP only out of DS IV.

Converse experiment: recombination of mCherry into EGFP-containing MVA

Recombination was performed with an mCherry shuttle plasmid into an already GFP-containing MVA-CR19 virus. The passage 1 plate was scanned after 3 days, and 7 red-only (r1 to r7) and 36 yellow foci were picked (out of a total of 1577 obtained foci, **Figure 5 (a)** first (P1) column). Within one passage, foci r2 and r5 out of the seven isolates already appeared to be R-R only. Extrapolating from the observed frequency of red signals after recombination with a parental green virus, a simultaneous insertion of inserts into both termini (which are separated by more than 170 kb) seems unlikely. The observation is more consistent with a mechanism by which both termini are aligned during replication and differences are then corrected towards identical copies probably within a single replication cycle.

Foci r1, r3 and r7 suggest coinfection with different viruses (parental green and newly formed red viruses), focus r4 with only EGFP signals may have been an erroneously picked G-G parental virus, a common complication in focus purification procedures.

Diluted preparations of the P1 foci r1 and r2 were used to infect a 6-well plate. Consistent with the genotypes, wells infected with r1 resulted in distinct mono-fluorescent red and green P2 foci whereas wells infected with r2 gave red-only P2 foci (**Figure 5 (a)** P2 columns for r1 and r2).

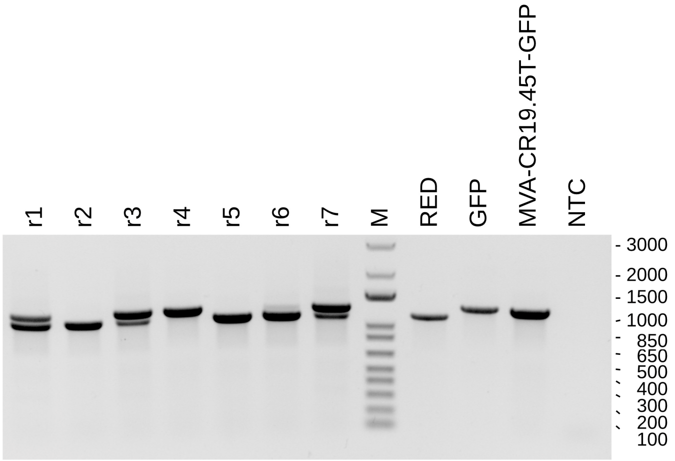

Figure S4. Genotype of P1 foci that were picked for a red phenotype in a replacement of an EGFP-1 expression cassette in MVA-CR19 against a mCherry expression cassette in a shuttle plasmid for DS IV. RED and GFP for reference PCRs with shuttle plasmids p4PT-RED and p45T-GFP as template, MVA-CR19.45T-GFP as positive control with viral genomic DNA, NTC as non-templare negative control. Expected amplicons are 1099 bp for DS IV containing mCherry, 1231 bp for DS IV containing GFP, approx. 2000 bp for a combination of both reporter genes, and 220 bp for an empty (WT) DS IV (see also Supplementary Table S1).

A bias towards co-infection

A bias towards co-infection is even seen in comet assays for which cultures are incubated without semi-solid medium [23]. The foci shown in **Figure S5** were obtained by infection of cell monolayers in 6-well plates with 10 PFU of preparations derived of the mixed isolate P2 y01* (of Figure 5) and the yellow isolate P4 y1 (Figure 3(a)) and. While the consistent yellow phenotype is clearly quantifiable for foci under semi-solid medium (8 of 8 foci are yellow), freely diffusable infections result in comets with yellow tails also for the isolate that shows a mixed phenotype under methylcellulose (16 green, 9 red, and therefrom 7 yellow foci).

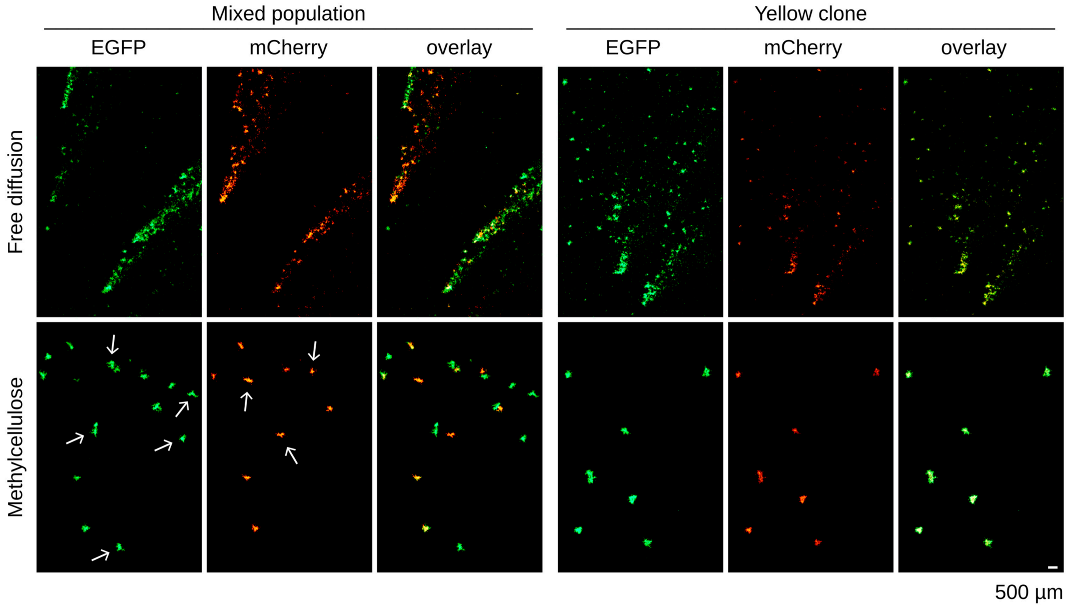

Figure S5. Separation of GFP and mCherry signals in a mixed population but not in the clone containing the single large insert. Both, comets and methylcellulose-confined foci show frequent separation into monofluorescent colors in the population with the mixed genotype (some highlighted with arrows in the panels for EGFP and mCherry). The yellow clone shows only congruent fluorescence for both colors.

Experimental schematic of tetherin-assisted recombination

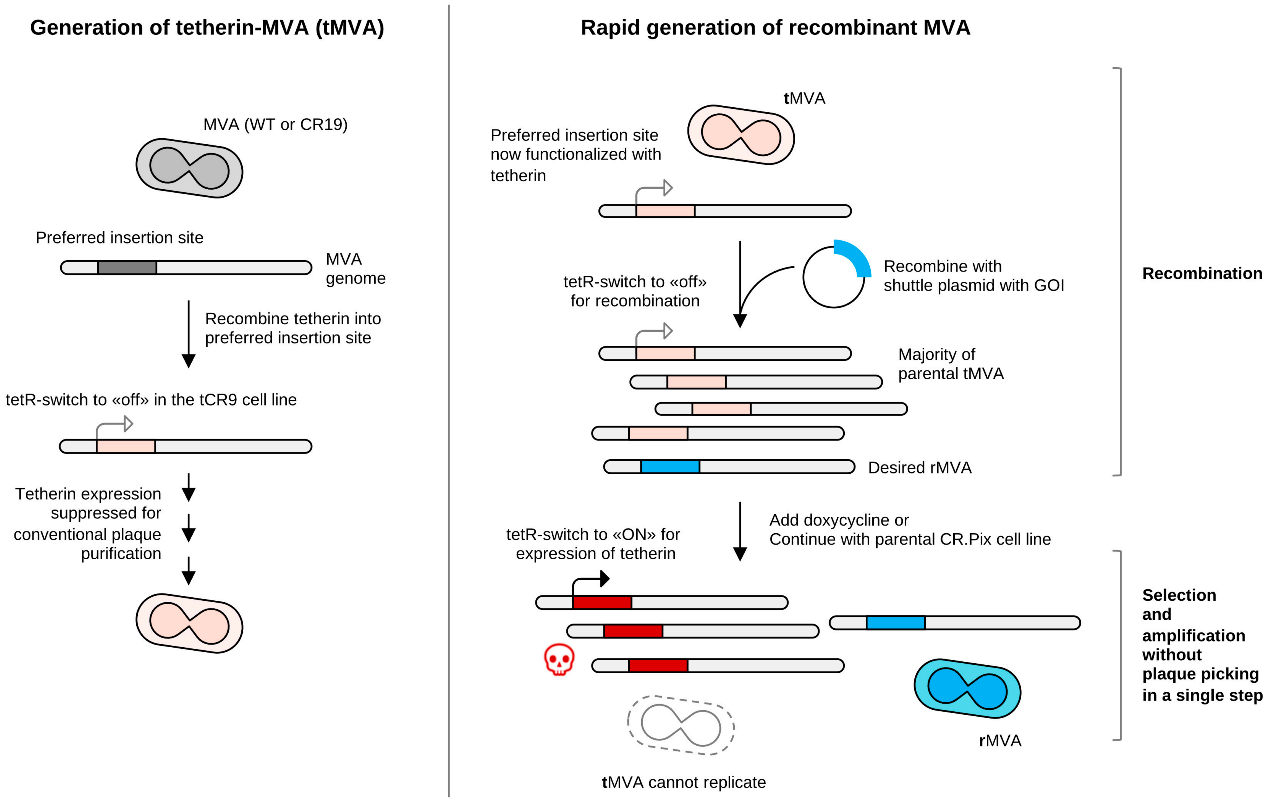

Figure S6. Tetherin-assisted generation of recombinant MVA (rMVA). The recipient tetherin-encoding virus (tMVA) is produced by conventional plaque purification in tCR9 cells. The tCR9 cell line expresses the TetR repressor protein in the cytoplasm to repress marker transcription. The appropriate tMVA viruses can be generated and characterised in advance. For the actual generation of rMVA, the tMVAs are used for homologous recombination with a shuttle plasmid containing a gene of interest and recombination flanks that frame the insertion site of tetherin. As viral replication is a prerequisite for recombination, tetherin must remain suppressed during this first step. In the subsequent passage, the block on tetherin expression is lifted (either by the addition of doxycycline or by passage in parental CR.pIX cells) and only the desired rMVAs can replicate. Parental tMVA viruses are not obtained when tetherin expression is allowed and plaque picking is therefore not required.

Automated counting of foci after tetherin-assisted recombination

Marker replacement was performed with receiving viruses that express tetherin and mCherry. The shuttle plasmid contained homologous flanks to DS IV with an expression cassette for mCherry and EGFP. Homologous recombination therefore results in viruses that maintain mCherry expression and gain EGFP expression resulting in an equal amount of foci with red and green fluorescence, or a relative amount of 0.5 (50 %) in the total number of foci.

The experiment was performed with MVA-CR19 viruses that contain tetherin in DS IV under control of the HYBdx (Figure 8) and M52dx promoter.

A slight bias towards EGFP expression can be observed possibly because expression from the viral promoter for EGFP is shifted towards earlier time points relative to the late P11 promoter that drives mCherry.

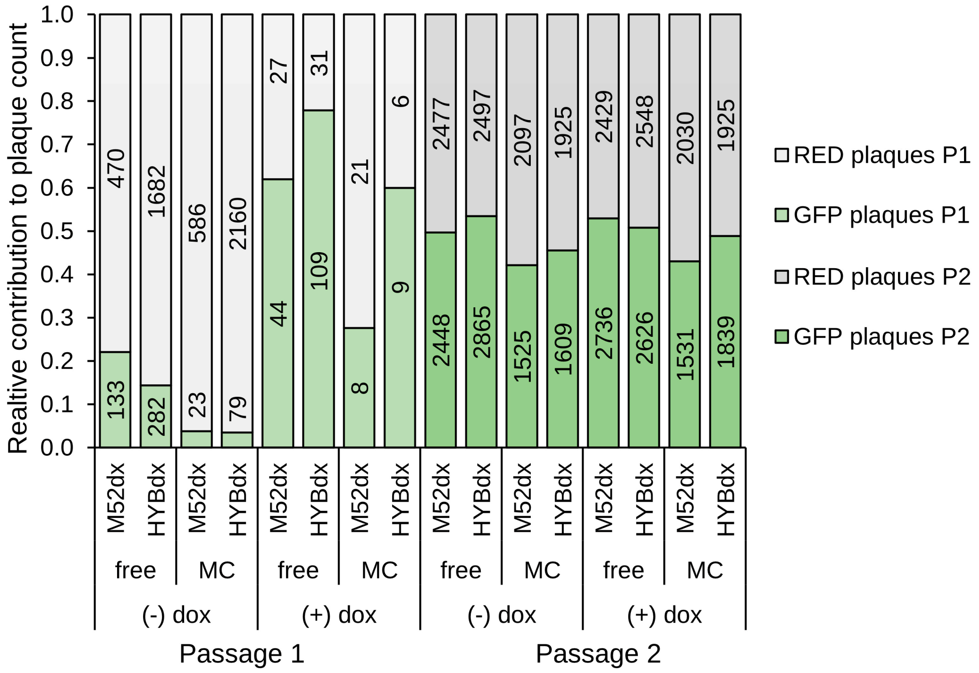

Figure S7. Tetherin expression cassette is replaced within one passage by the desired transgene. Tetherin expression is under control of the mH5dx or HYBdx promoters (mH5 or Hyb promoters with downstream tetO repeats). The desired result by automated quantification is an increase of EGFP expression towards a relative ratio of 0.5 if expression of tetherin is unblocked ((+)dox) as opposed to repressed ((-)dox). Quantification was performed in cultures under methylcellulose (MC) or normal medium for free diffusion of progeny virus.
